# Supplementary material for: A Novel Hybrid Membrane Process Coupled with Freeze Concentration for Phosphorus Recovery from Cheese Whey
Source: Membranes (Basel). 2023 Apr 21;13(4):450. doi: 10.3390/membranes13040450 (PMC10147047; doi:10.3390/membranes13040450)
Supplement: Supplementary file 1 [file membranes-13-00450-s001.zip › membranes-2308913-supplementary.pdf]

membranes

MDPI

Article

A Novel Hybrid Membrane Process Coupled with Freeze Concentration for Phosphorus Recovery from Cheese Whey

Ipan Hidayat [1], Lidia Paredes [1,*], Pablo M. Binder [1], Nagore Guerra-Gorostegi [1], Mabel Mora [1], Sergio Ponsá [1], Darren L. Oatley-Radcliffe [2] and Laia Llenas [1]

[1] BETA Technological Center (TECNIO Network), University of Vic—Central University of Catalonia (UVic-UCC), Carretera de Roda 70, 08500 Vic, Spain
[2] Energy Safety Research Institute (ESRI), College of Engineering, Swansea University, Bay Campus, Swansea, Wales SA1 8EN, UK
* Correspondence: lidia.paredes@uvic.cat

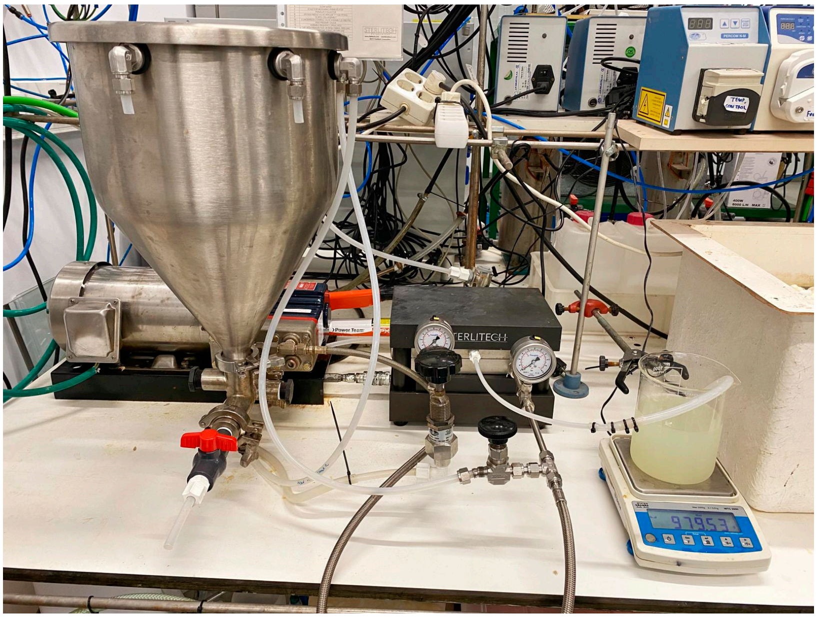

Figure S1. The real picture of apparatus used of membrane system.

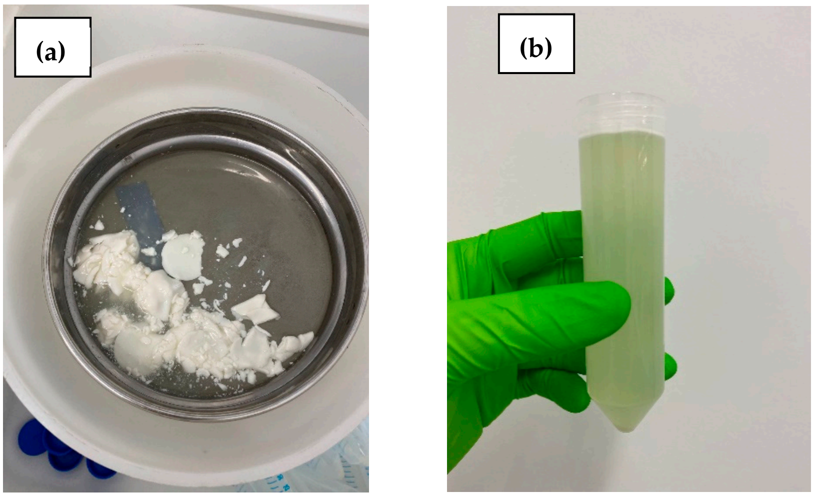

Figure S2. Precipitation following lactic acid acidification (a) and supernatant of the centrifugation process (b).

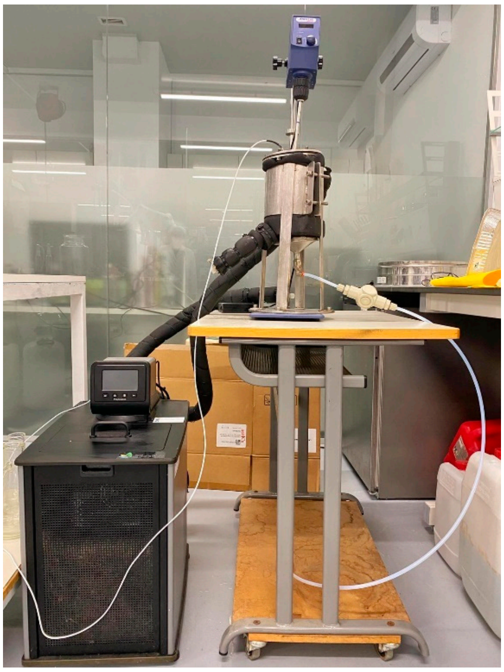

Figure S3. The real picture of apparatus used of progressive freeze concentration.

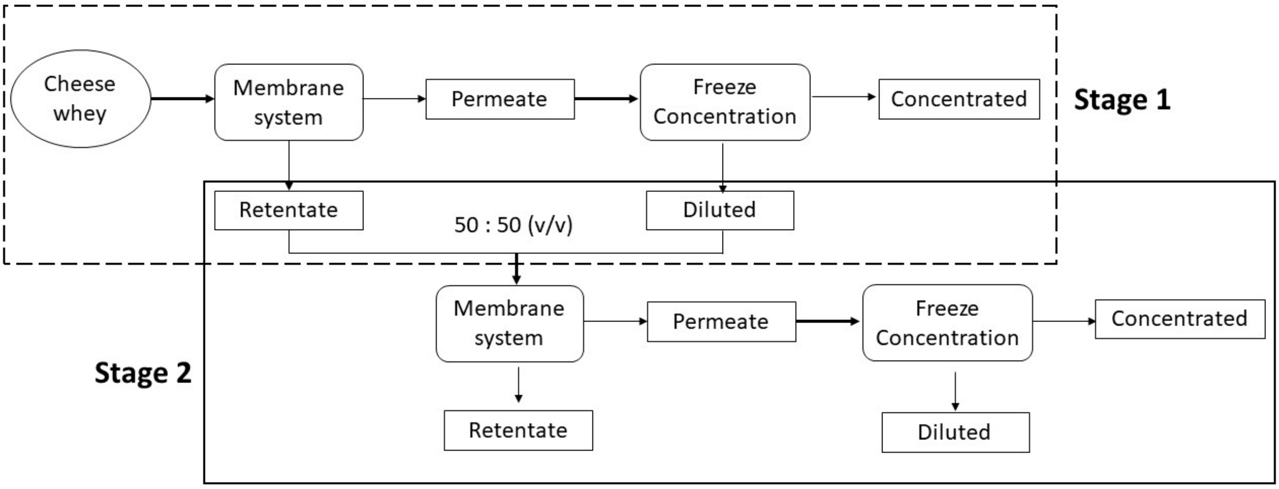

Figure S4. Flowchart of the two-stages of cheese whey valorisation using coupled technology of membrane system and freeze concentration.
